# Droplet Encoding‐Pairing Enabled Multiplexed Digital Loop‐Mediated Isothermal Amplification for Simultaneous Quantitative Detection of Multiple Pathogens

**DOI:** 10.1002/advs.202205863

**Published:** 2023-01-16

**Authors:** Dongyang Cai, Yu Wang, Jingjing Zou, Zhujun Li, Enqi Huang, Xiuyun Ouyang, Zhiquan Que, Yanzhang Luo, Zhenhua Chen, Yanqing Jiang, Guohao Zhang, Hongkai Wu, Dayu Liu

**Affiliations:** ^1^ Department of Laboratory Medicine the Second Affiliated Hospital School of Medicine South China University of Technology Guangzhou 510180 China; ^2^ College of Food Science and Engineering South China University of Technology Guangzhou 510640 China; ^3^ Beijing Baicare Biotechnology Co., Ltd Beijing 102206 China; ^4^ Department of Chemistry Hong Kong University of Science and Technology Hong Kong China; ^5^ Guangdong Engineering Technology Research Center of Microfluidic Chip Medical Diagnosis Guangzhou 510180 China; ^6^ Clinical Molecular Medicine and Molecular Diagnosis Key Laboratory of Guangdong Province Guangzhou 510180 China

**Keywords:** droplet encoding‐pairing, droplet microfluidics, machine learning algorithm, multiplexed droplet digital nucleic acid analysis, quantitative detection of pathogens

## Abstract

Despite the advantages of digital nucleic acid analysis (DNAA) in terms of sensitivity, precision, and resolution, current DNAA methods commonly suffer a limitation in multiplexing capacity. To address this issue, a droplet encoding‐pairing enabled DNAA multiplexing strategy is developed, wherein unique tricolor combinations are deployed to index individual primer droplets. The template droplets and primer droplets are sequentially introduced into a microfluidic chip with a calabash‐shaped microwell array and are pairwise trapped and merged in the microwells. Pre‐merging and post‐amplification image analysis with a machine learning algorithm is used to identify, enumerate, and address the droplets. By incorporating the amplification signals with droplet encoding information, simultaneous quantitative detection of multiple targets is achieved. This strategy allows for the establishment of flexible multiplexed DNAA by simply adjusting the primer droplet library. Its flexibility is demonstrated by establishing two multiplexed (8‐plex) droplet digital loop‐mediated isothermal amplification (mddLAMP) assays for individually detecting lower respiratory tract infection and urinary tract infection causative pathogens. Clinical sample analysis shows that the microbial detection outcomes of the mddLAMP assays are consistent with those of the conventional assay. This DNAA multiplexing strategy can achieve flexible high‐order multiplexing on demand, making it a desirable tool for high‐content pathogen detection.

## Graphical abstract

The developed droplet encoding‐pairing enabled multiplexed droplet digital nucleic acid analysis strategy, which can be adapted to any amplification methods, features high flexibility in constructing high‐order multiplexed droplet digital assays on demand, thus providing a desirable tool for simultaneous quantitative detection of complex and mutative infection‐causing pathogens.

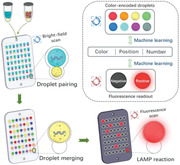

## 1 Introduction

Digital nucleic acid analysis (DNAA) achieves absolute quantification of target nucleic acids by dividing a sample into large amounts of miniaturized compartments, followed by Poisson statistical analysis of the end‐point binary readouts from each compartment.^[^ ^1^, ^2^ ^]^ By implementing this “divide and conquer” strategy, DNAA transforms fractional cycle analysis of exponential amplification profiles into a digital counting format, eliminating the need for standard curves.^[^ ^3^, ^4^ ^]^ Moreover, nucleic acid confinement in such miniaturized compartments dramatically increases the effective molecule concentration and isolates target nucleic acids from interfering substances (e.g., amplification inhibitors and/or off‐targets), thus providing a higher detection sensitivity, precision, and resolution compared to conventional bulk‐volume NAA.^[^ ^5^, ^6^ ^]^ Benefiting from these merits, DNAA has been increasingly deployed in the quantitative detection of pathogens in clinical samples.^[^ ^7^, ^8^ ^]^

Despite the aforementioned advantages, current DNAA methods commonly suffer a limitation in multiplexing capacity.^[^ ^3^ ^]^ This limitation is particularly evident in pathogen detection applications, wherein an infection often involves multiple candidate pathogens and therefore multiplexed detection capabilities are critical for prompt evidence‐based decision‐making. Various attempts have been made to overcome this limitation.^[^ ^9^ ^]^ The most commonly used multiplexing strategies by commercial DNAA platforms can be divided into three categories: i) color‐based multiplexing, in which multicolor fluorophore‐labeled probes are used to differentiate each amplicon.^[^ ^10^, ^11^ ^]^ However, its multiplexing capacity is limited by available fluorescence channels in the reader (e.g., Bio‐rad QX100/200 Droplet Digital PCR systems with two fluorescence channels) and the spectral overlap of the fluorophores; ii) amplitude‐based multiplexing, which is achieved by varying primer/probe concentrations^[^ ^12^, ^13^ ^]^ or amplicon length^[^ ^14^, ^15^ ^]^ to regulate the end‐point fluorescence intensity for each droplet cluster; and iii) probe mixing‐based multiplexing, wherein probes mixed at designated ratios are leveraged to create a unique fluorescent signature for each droplet cluster.^[^ ^16^, ^17^ ^]^ However, the latter two strategies require extensive optimization and troubleshooting process in thermodynamic efficiencies and/or chemical kinetics.^[^ ^18^ ^]^ Moreover, the separation of droplet clusters might not be distinct enough and therefore hampers accurate quantification.^[^ ^19^ ^]^

More recently, several custom methods for multiplexed DNAA have been developed. For instance, analysis of amplification profiles,^[^ ^20^, ^21^ ^]^ melting curves,^[^ ^22^, ^23^, ^24^, ^25^, ^26^ ^]^ or their integration^[^ ^27^, ^28^ ^]^ of each compartment can achieve multiplexed DNAA. These methods effectively expand the multiplexing capacity of DNAA. However, extensive optimization is needed to obtain amplicons with specific amplification profiles/melting curves and balance the amplification efficiencies due to the co‐existence of all primer sets in each compartment. Moreover, dedicated instrumentation is needed for real‐time monitoring of each compartment. Padlock probes, each containing a specific ratio of binding sites for two molecular beacons conjugated with different fluorophores, have been used in multiplexed digital rolling cycle amplification.^[^ ^18^ ^]^ This method has the potential for unlimited multiplexing capacity. However, misleading results may occur when a droplet contains more than one target molecule. Several studies on microfluidic chip‐based multiplexing strategies have also been reported, in which multiple DNAA assays, each detecting a specific target, were performed in independent sections.^[^ ^8^, ^29^, ^30^ ^]^ Although this method is simple and convenient for expanding the multiplexing capacity, it lacks flexibility in arbitrary high‐order multiplexing capacity without redesigning a new chip. Therefore, there remains an unmet need for developing a multiplexed DNAA strategy with high flexibility in achieving high‐order multiplexing on demand.

Droplet microfluidics features high flexibility in droplet manipulation, such as droplet encoding, trapping, pairing, and merging, making it a desirable platform for expanding the DNAA multiplexing capacity.^[^ ^31^ ^]^ A unique code as an identifier can be co‐encapsulated with each experimental element into a droplet and used to index the droplet individually in a droplet library.^[^ ^32^, ^33^ ^]^ This droplet encoding operation is especially beneficial to high‐throughput screening. A microfluidic chip patterned with particular geometries can be used to accommodate one or multiple droplets, thus pairing. Droplet trapping enables droplet addressing, pairing, and monitoring of droplet reaction signals over time.^[^ ^34^ ^]^ Merging of paired droplets from different droplet libraries (usually a sample droplet library and a reagent droplet library) allows their contents to mix. This process can efficiently map out the combinatorial space of pairwise interactions between two libraries without requiring massive operations.^[^ ^31^ ^]^ Accordingly, highly multiplexed and scalable detection assays have been developed by deploying these droplet manipulation technologies. For instance, Ackerman et al. developed the CARMEN platform (also proposed bCARMEN) and demonstrated its applications for high‐throughput pathogen detection.^[^ ^35^, ^36^ ^]^ However, the inevitable pairing of droplets from the same library (i.e., sample‐sample and reagent‐reagent droplet pairing) in a symmetric figure‐of‐eight microwell resulted in a substantial proportion (≈50%) of invalid reaction units. These false blank units artificially increase the proportion of “empty” units, which is the basis of Poisson distribution‐based digital quantification. Consequently, a scheme that permits high‐efficient sample‐reagent droplet pairing is desired for developing multiplexed DNAA.

**Figure 1:**
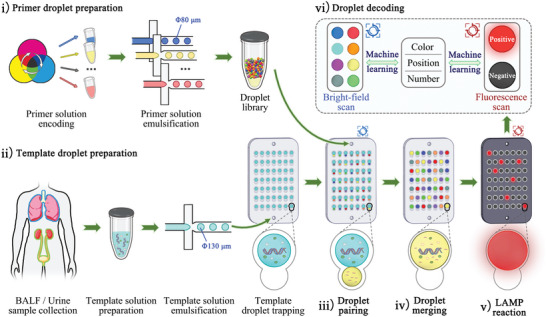
Schematic illustration of the workflow of the droplet encoding‐pairing enabled mddLAMP assay. i) Tricolor dyes were combined to generate multiple color codes. Each of the primer sets, along with a unique color code, was subjected to a droplet generator to form the primer droplets. Various primer droplet sets were pooled as designated to form a droplet library; ii) The template solution was emulsified to form template droplets, which directly flowed into the microfluidic chip and then trapped in the large sub‐wells. An aliquot of pooled primer droplets was injected into the same chip and the droplets were trapped in the small sub‐wells, thus forming primer‐template droplet pairs; iii) Bright‐field imaging after droplet pairing to identify, enumerate, and address the primer droplets; iv) The template droplets merged with the primer droplets to form a multiplexed DNAA reaction system; v) Fluorescence imaging after the LAMP reaction to identify, enumerate, and address the positive droplets; vi) Mapping the positive droplets back to the color‐encoded primer droplets to determine the identity and proportion of the positive droplets. *F*_b_ is the buoyancy force.

In this study, we developed a droplet encoding‐pairingenabled microfluidic DNAA multiplexing strategy. As shown in **Figure** **1** and Figure S1, Supporting Information, biocompatible dyes in tricolor of pigment were combined to generate multiple visual colors with distinct RGB (red, green, blue) values, which were used for primer droplet encoding. A microfluidic chip with a calabash‐shaped microwell array was used to sequentially and size‐selectively trap large‐volume template droplets and small‐volume primer droplets. The high‐density microwell array on the microfluidic chip permits the spontaneous formation of all possible combinations of the template and primer droplets through random droplet pairing. This spatially confined droplet manipulation eliminates the invalid droplet pairing of the sample–sample and reagent–reagent. As a result, the distribution of each target molecule across the entire droplet array follows the Poisson distribution. A machine learning algorithm was used to identify, enumerate, and address primer droplets from bright‐field imaging and positive droplets from fluorescence imaging. By incorporating the amplification signals with droplet encoding information, highly multiplexed DNAA was achieved. This multiplexing strategy features high flexibility in developing high‐order multiplexing on demand, which can be achieved by simply adjusting the number and/or types of input primer droplet sets (i.e., the content of a droplet library).

We demonstrate the flexibility of this DNAA multiplexing strategy by establishing two multiplexed droplet digital loop‐mediated isothermal amplification (mddLAMP) assays for detecting the pathogens responsible for lower respiratory tract infections (LRTIs) and urinary tract infections (UTIs), respectively. Clinical sample analysis shows that the microbial detection outcomes of the mddLAMP assays were consistent with those of the conventional assay. This DNAA multiplexing strategy makes it simple to establish flexible multiplexed DNAA assays on demand, thus providing a desirable tool for high‐content pathogen detection.

## 2 Experimental Section

### 2.1 Microfluidic Chip Design and Fabrication

The mddLAMP assay consisted of two sub‐component chips: i) droplet generators in flow‐focusing geometries (Figure S2, Supporting Information) for generating large‐volume template droplets with a diameter of 130 µm and small‐volume primer droplets with a diameter of 80 µm (Figure S3, Supporting Information); and ii) a calabash‐shaped microwell array chip (10 000 and 50 000 microwells) for droplet trapping, pairing, and merging. Both chips were made of polydimethylsiloxane (PDMS) by standard soft lithography techniques. After plasma treatment, a bottom layer with a channel (300 µm in depth for droplet floating and spreading) and a top layer with calabash‐shaped microwells were bonded to form the microwell array chip. Each calabash‐shaped microwell consisted of two cylindrical structures (a smaller one with a diameter of 90 µm and a depth of 65 µm; a larger one with a diameter of 135 µm and a depth of 110 µm) with 15 µm overlap along the upper circular base. The microwells were arrayed in a lattice pattern with 50 µm inter‐well spacing. Access holes were punched on the top layer to make the inlet and outlet for droplet injection and flushing away excess droplets. After fabrication, the droplet generators and microwell array chips were heated at 120 °C for 8 h to restore hydrophobicity.

### 2.2 Standard Bacterial Strains and Clinical Samples

The standard bacterial strains used in this study included *Streptococcus pneumoniae* ATCC 49619, *Pseudomonas aeruginosa* ATCC 27853, *Escherichia coli* ATCC 25922, *Staphylococcus aureus* ATCC 29213, *Acinetobacter baumannii* ATCC 19606, *Moraxella catarrhalis* ATCC 25238, *Legionella pneumophila* ATCC 33152, *Haemophilus influenzae* ATCC 49247, *Enterococcus faecalis* ATCC 29212, *Klebsiella pneumoniae* ATCC 700603, *Proteus mirabilis* CMCC 49005, and *Streptococcus agalactiae* CICC 10465*. H. influenzae* ATCC 49247 was overnight cultured on chocolate agar plates under 5% CO_2_, and other strains were overnight cultured on blood agar plates.

The bronchoalveolar lavage fluid (BALF) and urine samples submitted to the clinical microbiology laboratory of Guangzhou First People's Hospital were processed for routine testing, including Gram‐staining, quantitative bacterial culture, and antimicrobial susceptibility testing. Remnant BALF and urine samples were stored at −80 °C within 24 h of collection. All sample collections were approved by the Ethics Committee of the Guangzhou First People's Hospital (IRB: K‐2021‐127‐01 and K‐2021‐057‐01) and the study was conducted in accordance with relevant guidelines. BALF samples with colony counts more than 10^4^ CFU·mL^−1^ and urine samples with colony counts more than 10^5^ CFU·mL^−1^ were deemed significant infection, and those with lower colony counts were assumed to be contamination or colonization.^[^ ^37^, ^38^, ^39^ ^]^

### 2.3 DNA Extraction

For DNA extraction from standard bacterial strains, a single colony (with the exception of *P. mirabilis* CMCC 49005 due to its swarming growth on blood agar) from each plate was added to 100 µL of QuickExtract DNA Extraction Solution (Epicentre). The samples were incubated at 65 °C for 15 min and 98 °C for 2 min. The extracted genomic DNA (gDNA) samples were stored at −80 °C until use. A 4‐fold dilution series of a contrived sample containing eight different types of bacterial gDNA was prepared in 1 mg·mL^−1^ BSA (Sigma).

For DNA extraction from clinical samples, an aliquot of remnant sample (2 mL) was pelleted at 8000 g for 10 min. The bacterial pellet was then resuspended in a 100‐µL solution containing 1 µL of DNase I (New England Biolabs) and 10 µL of DNase I Reaction Buffer (New England Biolabs). After incubation at 37 °C for 10 min, the sample was pelleted at 8000 g for 10 min. This DNase treatment was used to degrade extracellular DNA present in the samples to prevent the overestimation of bacterial density. After discarding the supernatant, an aliquot of QuickExtract DNA Extraction Solution (100 µL for BALF and 200 µL for urine) was added to the sample. DNA extraction was performed as described above. This DNA extraction process resulted in a 20‐fold enrichment in bacterial gDNA concentrations for BALF samples and a 10‐fold enrichment for urine samples.

### 2.4 Preparation of Template Droplets and Color‐Encoded Primer Droplets

A 1‐mL plastic syringe affixed with Teflon tubing (i.d. 0.46 mm, o.d. 0.92 mm) was filled with HFE‐7500 oil containing 2% (wt/wt) 008‐FluoroSurfactant (RAN biotechnologies). Another syringe affixed with Teflon tubing was filled with pure HFE‐7500 oil (3 M), and a 50‐µL template solution (see detailed components below) was loaded into the tubing. These two syringes were directedly connected to the corresponding droplet generator via the Teflon tubing and then actuated with a syringe pump (Biotaor). The generated template droplets directly flowed into the oil‐filled microwell array chip (RAN biotechnologies) via Teflon tubing.

To generate the color code for primer droplets, three indicator dyes including phenol red (PR, Sigma), bromothymol blue (BTB, Sigma), and orange G (OG, Sigma) were selected. These indicator dyes were individually dissolved in nuclease‐free water (Takara) at a concentration of 10 mM. Combinations of two‐indicator dyes were prepared according to the volume ratios in Table S1, Supporting Information (also see corresponding primer sets). A volume of 10.6 µL of these dye combinations was added to the primer solutions (see detailed components below). The generation of primer droplets was as described above. The primer droplets were collected in a PCR strip. A volume of 10 µL of each primer droplet set was pooled into a single PCR tube via pipetting to form a droplet library. The tube was manually flipped several times to mix the droplets thoroughly.

### 2.5 Droplet Digital LAMP

The template droplet contained the following components: 1× isothermal amplification buffer (New England BioLabs), additional 4.8 mM MgSO_4_ (1.2×, New England BioLabs), 384 U·mL^−1^ *Bst* 2.0 (1.2×, New England BioLabs), 1.68 mM dNTPs (1.2×, New England BioLabs), 0.96 M betaine (1.2×, Sigma), 1.2 mg·mL^−1^ BSA (1.2×, Sigma), 2.4 µM SYTO 82 (1.2×, ThermoFisher), 1× Ambion RNase cocktail (Invitrogen), and varying amounts of DNA template (10 µL for standard bacterial strains and BALF samples and 2 µL for urine samples in 50 µL template solution). The RNase cocktail was used to eliminate a potential source of error caused by the reverse transcription activity of *Bst* DNA polymerase.^[^ ^40^ ^]^

The primer droplet contained the following components: 1× isothermal amplification buffer (New England BioLabs), 2.12 mM dye combinations (5.3×), and primer premix: 5.3× (1.6 µM FIP/BIP, 0.2 µM F3/B3, and 0.8 µM LF/LB). The primer sets used in the LRTI detection panel were kindly provided by Baicare Biotechnology Limited, while the primer sets used in the UTI detection panel were selected from the literature (Table S2, Supporting Information). After droplet merging, the final concentration of each LAMP component would be 1×. To perform LAMP, the microwell array chip was submerged in a 68 °C water bath for 60 min.

Due to the inability of this LAMP temperature to unwind all gDNA, not every target can be detected.^[^ ^8^, ^41^, ^42^ ^]^ To increase the detection sensitivity, the DNA template was preheated at 95 °C for 5 min and immediately cooled on ice for 3 min to obtain single‐stranded DNA (ssDNA). Subsequently, 20 µg·mL^−1^ ET SSB (New England BioLabs) was added to stabilize the ssDNA during droplet emulsification at ambient temperature (Figure S4, Supporting Information).

### 2.6 Droplet Trapping, Paring, and Merging

After manually tilting the microwell array chip, the template droplets spread and floated into the large sub‐wells by buoyancy force. Once all of the large sub‐wells were occupied, excess template droplets were flushed away from the outlet of the chip. Afterward, the pooled primer droplets (10 µL for the small‐dimension microwell array and 50 µL for the large one) were injected into the chip via pipetting and then floated into the small sub‐wells, forming random pairings with the previously trapped template droplets. After flushing away the untrapped primer droplets, a bright‐field image of the droplet array was captured. Subsequently, a lighter piezoelectric ignitor was used to apply an electrical field to merge the paired droplets (Figure S1, Supporting Information).

### 2.7 Droplet Array Imaging

All images were captured using a Leica DMi8 inverted fluorescence microscope equipped with a motorized stage and a Leica DFC7000 T camera. The microwell array was scanned at 5× magnification. For bright‐field imaging of color‐encoded primer droplets, the illumination settings were intensity 100, aperture 3, and TL‐Fld 46; the camera settings were exposure time 1 ms, digital gain 1.0, saturation 33, and white balance: R 3.1, G 0.0, B 14.8. After the LAMP reaction, the microwell array was rescanned to generate bright‐field and fluorescence images without moving the chip. The parameters for fluorescence imaging were 500 ms (exposure time), 555 nm (excitation wavelength), and 590 nm (emission wavelength). These three images, including the bright‐field image of paired droplets and the bright‐field and fluorescence images of merged droplets after the LAMP reaction, were subsequently stitched together using the Leica Application Suite X software (Leica).

### 2.8 Image Analysis with a Machine Learning Algorithm

For the bright‐field image of paired droplets, all droplet pairs were first recognized and numbered to acquire the position information. Following image segmentation, individual droplet pairs were extracted. The individual droplet pairs were then categorized into eight groups based on the color codes (i.e., RGB values) of the primer droplets. For the bright‐field image of merged droplets after the LAMP reaction, all droplets were recognized and their coordinates and sizes were recorded. For the fluorescence image of merged droplets after the LAMP reaction, the recorded coordinate and size data were used to recognize and address each droplet. Following image segmentation, individual droplets were extracted. The images were then converted from RGB to HSB (Hue, Saturation, Brightness) space, and the B values were used to identify positive droplets. By mapping the positive droplets back to the color‐encoded primer droplets, the identity and proportion of positive droplets were determined (Figure S5, Supporting Information).

### 2.9 Digital PCR

The mddLAMP was compared with the OsciDrop, a commercial digital PCR system (Dawei Biotech, approved by the China National Medical Products Administration). The digital PCR reaction mixture (25 µL) contained the following components: 12.5 µL of 2× dPCR SuperMix (Dawei Biotech), 0.6 µL of DNA polymerase (Dawei Biotech), 2.5 µL of primer mix (10 mM, B3/F3 primers from the LAMP primer sets were used in digital PCR), 1.25 µL EvaGreen (Biotium), 2 µL of template DNA, and 6.15 µL of nuclease‐free water (Takara). The thermocycling protocol was as follows: 95 °C for 5 min, followed by 45 cycles of 95 °C for 20 s and 56 °C for 1 min.

### 2.10 Data Analysis

All image analysis was performed with the machine learning algorithm in Python 2.7 installed on a common laptop. The adaptive threshold algorithm detected the profile of the droplets. The *k*‐nearest neighbor algorithm categorized the primer droplets into different groups based on their RGB values. Afterward, a custom position reasoning and line‐fitting algorithm were used to obtain the position of the droplets. The blink‐detection algorithm then identified positive droplets. Finally, the Openpyxl module recorded the color, number, and position data of the droplets. For more information, please see the provided code and package documentation.

The mathematical models in Figure 4A,B were plotted with the NumPy package and Matplotlib documentation in Python 3.8. The precise formulas used can be found in reference 49.

## 3 Results

### 3.1 Color Encoding of Primer Droplets

**Figure 2:**
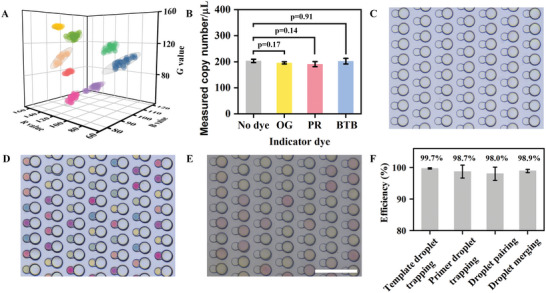
Droplet encoding, trapping, pairing, and merging. A) Distributions of the color codes in the RGB space with 95% confidence ellipses (*n* = 30 for each cluster); B) Influence of the indicator dyes (400 µM) on the quantification precision of the mddLAMP assay; C–E) Microphotographs of droplet trapping, pairing, and merging; F) Efficiencies for droplet trapping, pairing, and merging. (*n* = 3 technical replicates, scale bar = 500 µm).

Tricolor dye combinations were used to encode primer droplets, with one color corresponding to one primer set. Three chemically stable dyes, PR, BTB, and OG, were selected to construct the color code. PR and BTB are widely used as indicator dyes in colorimetric LAMP,^[^ ^43^, ^44^ ^]^ whereas OG is a commonly used tracing dye in gel electrophoresis.^[^ ^45^ ^]^ The colors of PR, BTB, and OG at pH 8.8 (i.e., the initial pH value of the LAMP reaction) are similar to magenta, cyan, and yellow, respectively, which is consistent with the tricolor of pigment. We demonstrate this encoding strategy by screening out eight colors with distinct RGB values to encode the primer droplets. Their distributions in the color space (with 95% confidence ellipses, *n* = 30 for each cluster) are shown in **Figure** **2**A. No overlap in the RGB values was observed among these color codes, indicating that a unique identifier without mutual interference was co‐encapsulated into the primer droplets. Compared to colorimetric LAMP which commonly uses 50–100 µM indicator dyes to visualize the amplification results,^[^ ^46^ ^]^ increased concentrations of dyes were needed to clarify the color code due to the extremely short optical path (80 µm in maximum) of the primer droplets. The influence of these three indicator dyes with a concentration up to 400 µM (i.e., the final concentration in merged droplets; *E. coli* gDNA as the target) on the quantification precision was evaluated. As shown in Figure 2B, there was no statistically significant difference (*p* ≥ 0.05, two‐tailed Student's *t*‐test) between the quantification results with and without the addition of these indicator dyes.

### 3.2 Droplet Trapping, Pairing, and Merging

By sequentially introducing the large‐volume template droplets and small‐volume primer droplets to the microfluidic chip, the template‐primer droplet pairing spontaneously formed in the calabash‐shaped microwell array. The efficiency of each step, including droplet trapping, pairing, and merging, was quantified by characterizing the occupation of the calabash‐shaped microwells and the content of trapped droplets with a bright‐field scan over the entire microwell array.^[^ ^47^ ^]^ The sequential and size‐selective trapping of large‐volume template droplets and small‐volume primer droplets and the subsequent pairing are shown in Figure 2C,D and Movies S1 and S2, Supporting Information. The efficiencies for trapping template droplets and primer droplets were 99.7% and 98.7%, respectively, and that for productive droplet pairing (i.e., a template droplet with a primer droplet) was 98.0% (Figure 2F). This size‐selective droplet trapping prevents the trapping of infrequently coalescent template droplets (> 160 µm in diameter) and primer droplets (>100 µm in diameter). Merging of the paired droplets was achieved via applying an electrical field to the microwell array with a lighter piezoelectric ignitor (Figure 2E and Movie S3, Supporting Information; water‐saturated PDMS after incubation in a water bath made the microphotograph slightly darker with the same imaging settings), and the merging efficiency on the basis of productive droplet pairing was determined to be 98.9% (Figure 2F). The component concentrations in the primer droplets were 5.3× and those in the template droplets were 1.2×. Accordingly, the concentrations for each LAMP component would be 1× after droplet merging.

### 3.3 Decoding the Droplet Array with a Machine Learning Algorithm

**Figure 3:**
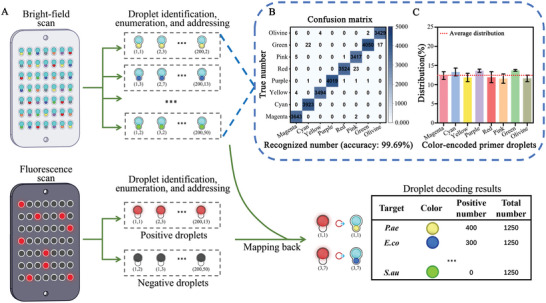
Decoding of the droplet array with a machine learning algorithm. A) Schematic illustration of the decoding workflow; B) Confusion matrix comparing the color classification results from the machine learning algorithm to artificial recognition (three experiment results combined); C) Distributions of the primer droplets with different color codes. The red dotted line indicates the theoretical mean (12.5%, *n* = 3 technical replicates).

Droplet decoding was performed with a custom machine‐learning algorithm. The decoding procedure included 4 steps: i) bright‐field image analysis of paired droplets to identify, enumerate, and address primer droplets; ii) bright‐field image analysis of merged droplets after amplification to record their coordinates and sizes. These data were later endowed to the corresponding fluorescence image since negative droplets were invisible after setting a fluorescence threshold, making it impossible to address the positive droplets; iii) fluorescence image analysis of merged droplets after amplification to identify, enumerate, and address positive droplets; iv) mapping the positive droplets back to the color‐encoded primer droplets (**Figure** **3**A and Figures S5 and S6, Supporting Information). The identity and proportion of positive droplets can be determined following this droplet decoding procedure. The confusion matrix comparing the color classification results from the machine learning algorithm to artificial recognition (considered as the true number) is shown in Figure 3B. The color recognition accuracy of the machine learning algorithm was 99.69% and the false recognition rate of each color was less than 1%. The decoding results also show that the number of primer droplets in each color code was nearly identically distributed (nearly 12.5% of all paired droplets, Figure 3C).

### 3.4 Simultaneous Quantitative Detection of Multiple Pathogens Using the mddLAMP Assay

**Figure 4:**
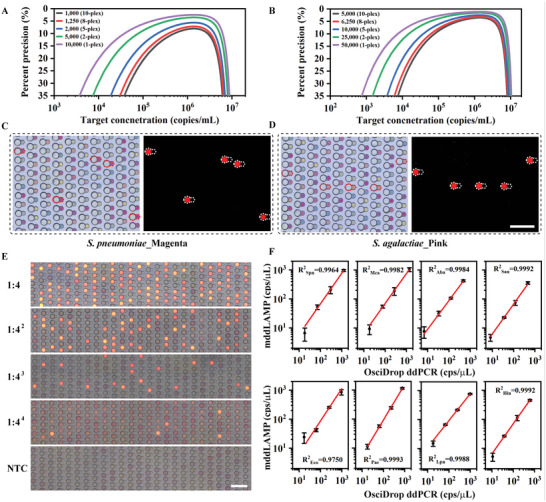
The mddLAMP assay for simultaneous quantitative detection of multiple bacterial nucleic acids. A,B) Mathematical models of measurement precision depending on the number of available partitions. The contour lines represent a specific level of precision (up to 35%) and associate the required partition number with the specified precision at various bacterial concentrations for the small‐dimension and large‐dimension microwell array chips, respectively; C,D) Representative bright‐field and fluorescence microphotographs showing the specificity of the LAMP primers used in the LRTI (*S. pneumoniae*) and UTI (*S. agalactiae*) detection panels against each input bacterial gDNA. Only the target corresponding merged droplets (dotted calabash‐shaped frame) showed increased fluorescence intensity; E) Bright‐field and fluorescence composite images showing a mddLAMP assay for detecting a 4‐fold dilution series of a contrived sample containing eight different types of bacterial gDNAs, NTC refers to no template control; F) Correlation of the mddLAMP quantification results with single‐plex dPCR quantification results. (*n* = 3 technical replicates, Scale bar = 500 µm)

As it stands, the developed mddLAMP assay has a trade‐off between multiplexing capacity and quantification precision, which depends on the number of available partitions. According to a guidance document, the acceptance criterion of relative repeatability standard deviation should be ≤ 35% across the entire dynamic range.^[^ ^48^ ^]^ We fabricated two chips with different dimensions in the microwell array (i.e., 10 000 and 50 000). As shown in **Figure** **4**A,B, the mathematical models^[^ ^49^ ^]^ indicate that the quantification precision decreases as the number of targets increases (from single‐plex to 10‐plex). However, the quantification precision using both chips for LRTI and UTI causative pathogen detection meets the acceptance criterion. Therefore, the small‐dimension chip was used in the following tests. To demonstrate the ability of the mddLAMP assay to simultaneously quantify multiple pathogenic nucleic acids, we tested a 4‐fold dilution series of a contrived sample containing eight different types of bacterial gDNA. No cross‐reaction was observed for any of the primer sets, demonstrating their high specificity (Figure 4C,D and Figure S7, Supporting Information). As shown in Figure 4E, the proportion of positive droplets decreased proportionally as the concentration of target DNA decreased. The mddLAMP results were compared to the single‐plex digital PCR results.^[^ ^50^, ^51^ ^]^ The quantification results reported by the mddLAMP assay and the commercial dPCR platform were in good agreement (Figure 4F).

### 3.5 mddLAMP for Analysis of Clinical Samples

To demonstrate the flexibility of this multiplexing strategy, we constructed two pathogen detection panels, one for LRTIs and one for UTIs, by simply modifying the primer droplet library. In each detection panel, the primer sets individually target the predominant infection‐causing pathogens. Specifically, the overlap in pathogen spectrum between LRTIs and UTIs (i.e., *P. aeruginosa*, *E. coli*, *S. aureus*, and *A. baumannii*) was encoded with the same color codes, while other infection‐specific targets (i.e., *S. pneumoniae*, *M. catarrhalis*, *L. pneumophila*, *and H. influenzae* for LRTIs; *E. faecalis*, *K. pneumoniae*, *P. mirabilis*, *and S. agalactiae* for UTIs) were distributed with different color codes.

**Figure 5:**
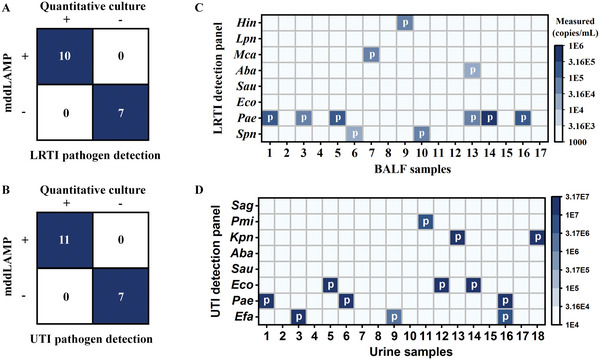
Detection of LRTI and UTI causative pathogens from 17 BALF and 18 urine samples using the mddLAMP in parallel with the quantitative bacterial culture. A,B) Concordance between the mddLAMP assay and the quantitative bacterial culture; C,D) Simultaneous quantitative detection of eight candidate pathogens with the LRTI and UTI detection panels, respectively. The heatmaps indicate the measured gDNA concentrations with the mddLAMP assay. The “*p*" symbol indicates the clinical outcomes obtained from the quantitative bacterial culture.

We tested 17 BALF and 18 urine samples with the mddLAMP in parallel with the conventional bacterial culture for quantitative detection of the LRTI and UTI causative pathogens. The turnaround time for testing a clinical sample with the mddLAMP assay is approximately 2.5 h (Table S3, Supporting Information). We determined the limit of blank (LOB) and limit of detection (LOD) of the LRTI and UTI detection panels based on the analysis of 10 NTCs (Table S4, Supporting Information, refer to the reference for precise formula).^[^ ^52^ ^]^ Tests yielding fewer positive droplets than the LOD were deemed negative and more positive droplets than the LOD suggested bacterial presence. As shown in **Figure** **5**A,B, seven BALF samples were determined as LRTI negative and another 10 samples as significant infection, meanwhile, seven urine samples were determined as UTI negative and another 11 samples as significant infection. The results reported by the mddLAMP assays are highly concordant with those reported by the quantitative bacterial culture. Of these positive samples, a BALF (sample 13) and a urine sample (sample 16) were determined as polymicrobial infection (targets within the detection panels), and the identification of the infection‐causing pathogens is also in good agreement with the outcomes of quantitative bacterial culture (*“p*” symbol in the box, Figure 5C,D and Table S5, Supporting Information).

## 4 Discussion and Conclusion

Considering that each systemic or localized infection involves multiple and variable pathogenic or opportunistic microorganisms, the detection panels for different infections need to be modified on demand. Following this requirement, we developed a droplet encoding‐pairing enabled multiplexing strategy, which features high flexibility in high‐order multiplexing capacity (Table S6, Supporting Information). First, visual colors resulting from tricolor dye combinations were used to encode primer droplets instead of spectroscopic labeling, eliminating the need for multiple fluorescence channels (e.g., the commercially available Luminex assay, in which different proportions of red and infrared fluorophores are employed to encode microspheres). Moreover, the color code does not interfere with the detection of reaction signals; Second, this multiplexing strategy deployed primer droplets of different colors, with each color corresponding to a specific target. Consequently, each droplet contained only a single set of primers/probes, eliminating the need for reaction system optimization; Third, the primer droplets for various targets were generated and stored individually, thus the pathogen detection panels for various infections can be easily constructed by modifying the composition of the primer droplet library on demand.

Moreover, the multiplexing strategy makes it simple to expand the DNAA multiplexing capacity. First, tricolor dye combinations can generate massive visual colors, thus this encoding strategy offers great expansibility in encoding capacity; Second, for a true color image with 24‐bit color depth, computers can display as many as 2^24^ different color combinations, which is the theoretical upper limit of the color code that can be recognized by the machine learning algorithm; Third, by using the calabash‐shaped microwell array, we achieved size‐selective droplet trapping that permits high‐efficient primer‐template droplet pairing. This random pairing process can spontaneously generate all possible combinations of primer droplets with template droplets, forming a comprehensive DNAA detection panel simultaneously.

In addition, the multiplexing strategy allows for the simultaneous quantitative detection of multiple targets with high precision. First, the machine learning algorithm recognizes the color code through the RGB value of a pixel. Thus, the difference in the three parameters profits the fidelity of the color code. Second, following the law of large numbers, the paired primer droplets in each color approach their theoretical mean of all paired droplets as the random pairing events increase (Figure 3C). Accordingly, the number of productive droplet pairs, which is the foundation of DNAA quantification precision, for each target detection can be increased by simply increasing the available array size. Third, the calabash‐shaped microwell array can prevent invalid droplet pairing (primer‐primer and template‐template pairing) and trapping of infrequently coalescent droplets, which may result in misleading results.

LRTIs and UTIs are associated with a variety of pathogens, and certain species of microorganisms are only determined as infection‐causing pathogens with colony counts over a defined cut‐off value, otherwise assumed to be colonized or contaminated. Consequently, simultaneous quantitative detection of multiple microorganisms is required to detect the causative pathogens. In this study, we developed two mddLAMP assays for individually detecting LRTI and UTI causative pathogens. This droplet encoding‐pairingenabled multiplexing strategy overcomes the difficulties in designing a multiplexed LAMP assay caused by its complex amplification chemistry. Clinical sample analysis shows that the microbial detection outcomes of the mddLAMP assays are consistent with those of the conventional assay, demonstrating the feasibility of the mddLAMP assay for clinical microbial detection.

Notably, this DNAA multiplexing strategy is also adaptable to other amplification methods, such as polymerase chain reaction, recombinase polymerase amplification, helicase‐dependent amplification, and rolling‐circle amplification. Future research will focus on increasing the array size and the number of input primer droplet sets to improve the detection throughput and dynamic range. If fully developed and validated for additional targets and sample types, this DNAA multiplexing strategy would facilitate timely and evidence‐based decision‐making and enhance disease intervention.

## Conflict of Interest

The authors declare no conflict of interest.

## Supporting information

Supporting Information

Supplemental Movie 1

Supplemental Movie 2

Supplemental Movie 3

## Data Availability

The data that support the findings of this study are available from the corresponding author upon reasonable request.
