# Supplementary material for: Droplet Encoding‐Pairing Enabled Multiplexed Digital Loop‐Mediated Isothermal Amplification for Simultaneous Quantitative Detection of Multiple Pathogens
Source: Adv Sci (Weinh). 2023 Jan 16;10(7):2205863. doi: 10.1002/advs.202205863 (PMC9982564; doi:10.1002/advs.202205863)
Supplement: Supplementary file 1 — Supporting Information [file ADVS-10-2205863-s002.pdf]

Supporting Information

Droplet encoding-pairing enabled multiplexed digital Loop-mediated isothermal amplification for simultaneous quantitative detection of multiple pathogens

*Dongyang Cai, Yu Wang, Jingjing Zou, Zhujun Li, Enqi Huang, Xiuyun Ouyang, Zhiquan Que, Yanzhang Luo, Zhenhua Chen, Yanqing Jiang, Guohao Zhang, Hongkai Wu, and Dayu Liu**

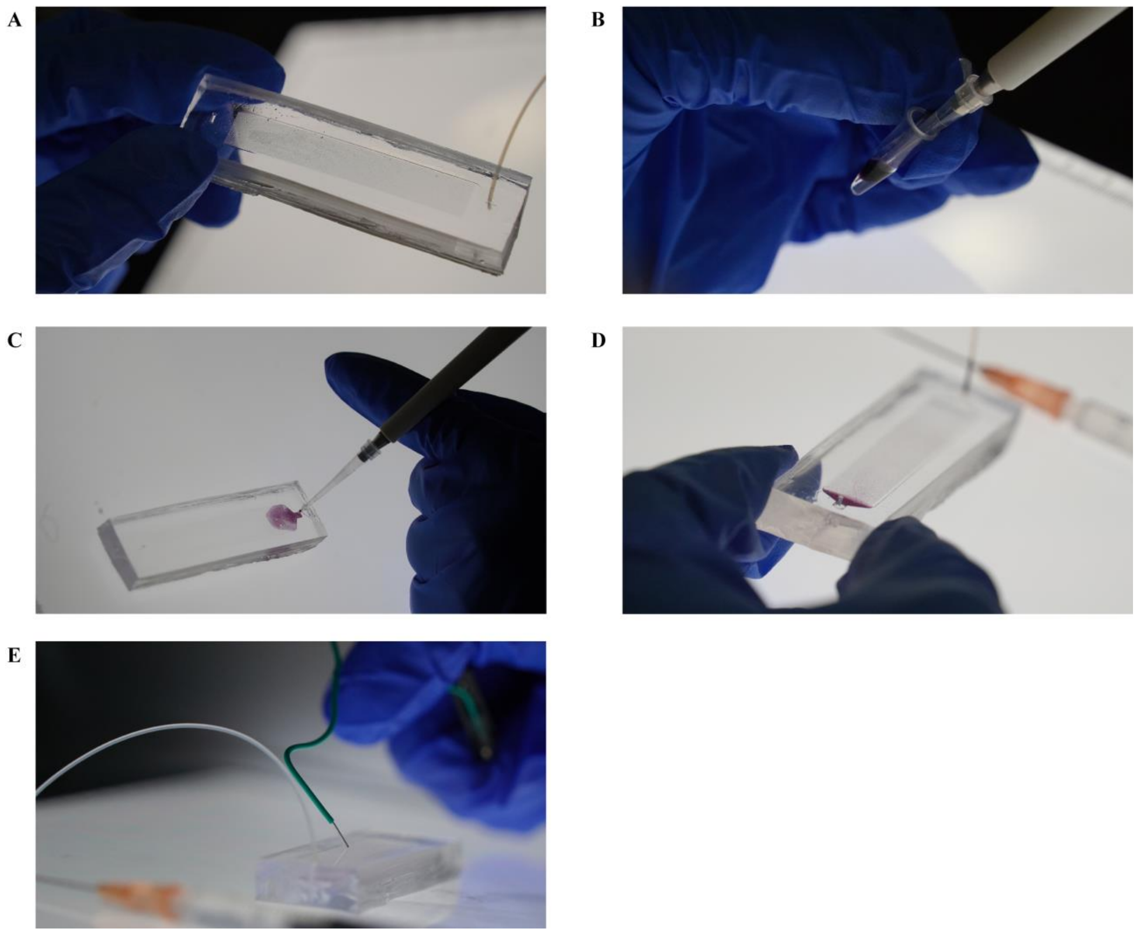

Figure S1. A) The generated template droplets directed flowed into the oil-filled microwell array chip via Teflon tubing. Afterward, the chip was manually tilted to capture the template droplets in the large sub-wells; B) Transfer of the pooled primer droplets via pipetting; C) Loading the color-encoded primer droplets into the microwell array chip; D) Manually tilting the microwell array chip to capture the primer droplets in the small sub-wells; E) Droplet merging with a lighter piezoelectric ignitor.

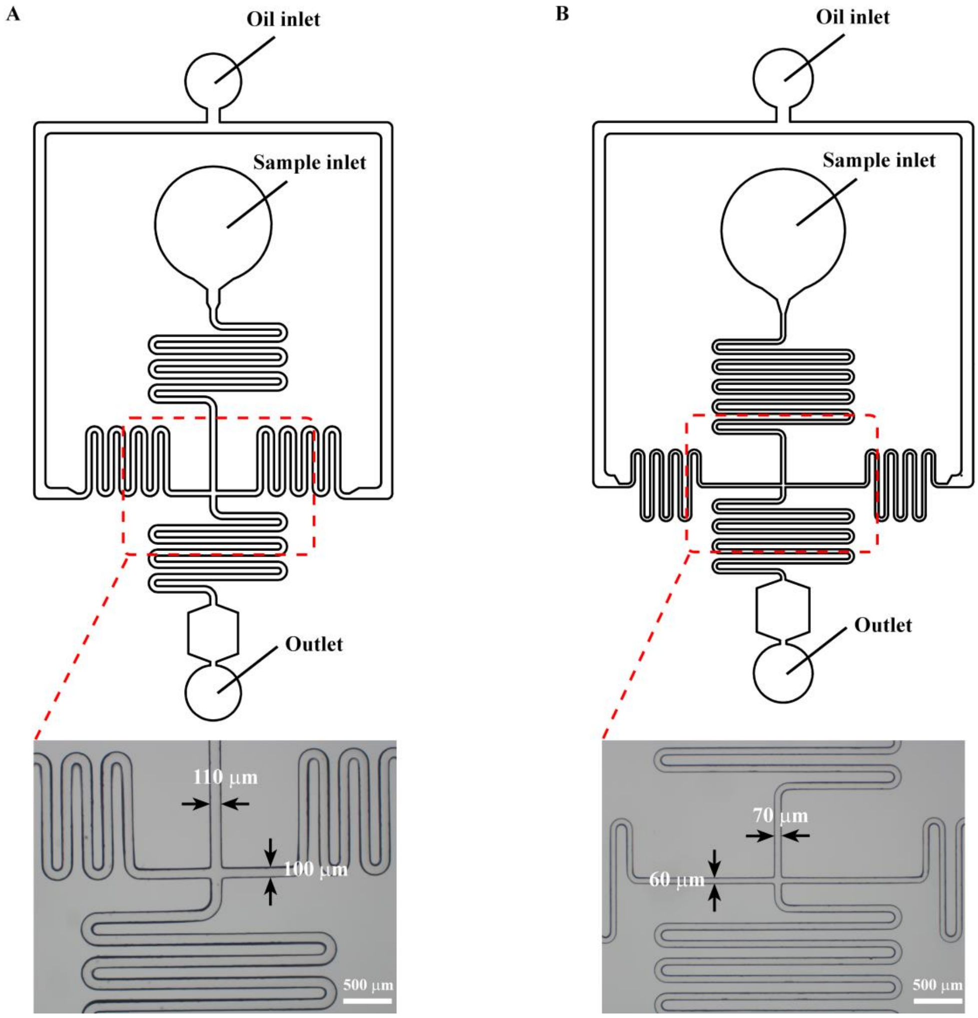

Figure S2. The CAD layouts (top view) of the droplet generators for producing the template droplets A) and primer droplets B) and the microphotographs of the flow-focusing zones. In the droplet generator for producing large-volume template droplets (130 μm in diameter), the sample channel is 110 μm in width, the oil channel is 100 μm in width, all the channels are 110 μm in height. The flow rate of the sample is 4 μL/min and that of oil is 14 μL/min. In the droplet generator for producing small-volume primer droplets (80 μm in diameter), the sample channel is 70 μm in width, the oil channel is 60 μm in width, all the channels are 50 μm in height. The flow rate of the sample is 5 μL/min and that of oil is 12 μL/min.

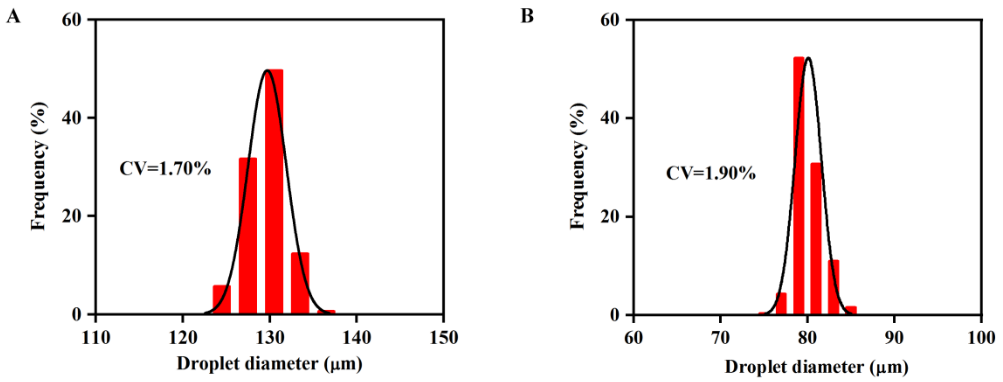

Figure S3. Size distribution of the generated template droplets and primer droplets. The average diameters for the template droplets and primer droplets were 129.9 μm with a CV of 1.70% and 80.1 μm with a CV of 1.90%, respectively (n=300, CV=coefficient of variation).

In this study, we found that using the mddLAMP assay for quantifying double-stranded bacterial gDNA is problematic: the quantification results using the mddLAMP were only ~15% of those achieved using ddPCR with the same sample. We speculate that DNA denaturation at 68 °C, which is much lower than the melting temperature of target DNA, was insufficient to unwind all DNA. LAMP primers cannot anneal to non-denatured DNA and thus not every DNA was detected. This problem is not vital in bulk-volume LAMP reaction because amplification of partial targets can also produce positive results although accompanied with compromised sensitivity. However, it results in “molecular dropout” in digital LAMP, which means a target is in present but does not amplify.[1] Therefore, preheating of dsDNA target together with the LAMP primers before the addition of DNA polymerase has been used in some digital LAMP studies to increase the detection sensitivity.[2–4] In our method, however, target DNA and LAMP primers were individually emulsified into different droplets and heating at denaturation temperature after droplet merging can inactivate the strand displacement DNA polymerase. Therefore, rapid cooling on ice after heat denaturation of DNA targets was used to obtain ssDNA and ssDNA binding protein was used to stabilize the obtained ssDNA during droplet emulsification at ambient temperature.

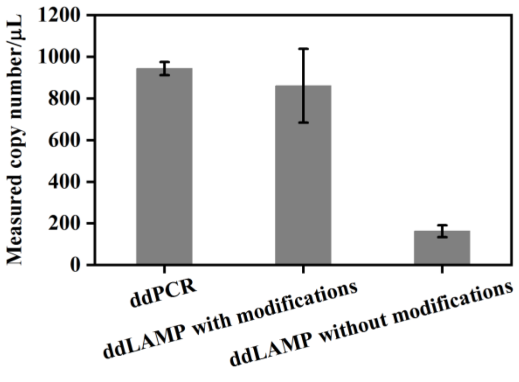

Figure S4. Evaluation of the influence of rapid cooling of heat denatured DNA and the addition of ET SSB protein (referred as modifications) on double-stranded DNA (*E. coli* gDNA) quantification with digital LAMP.

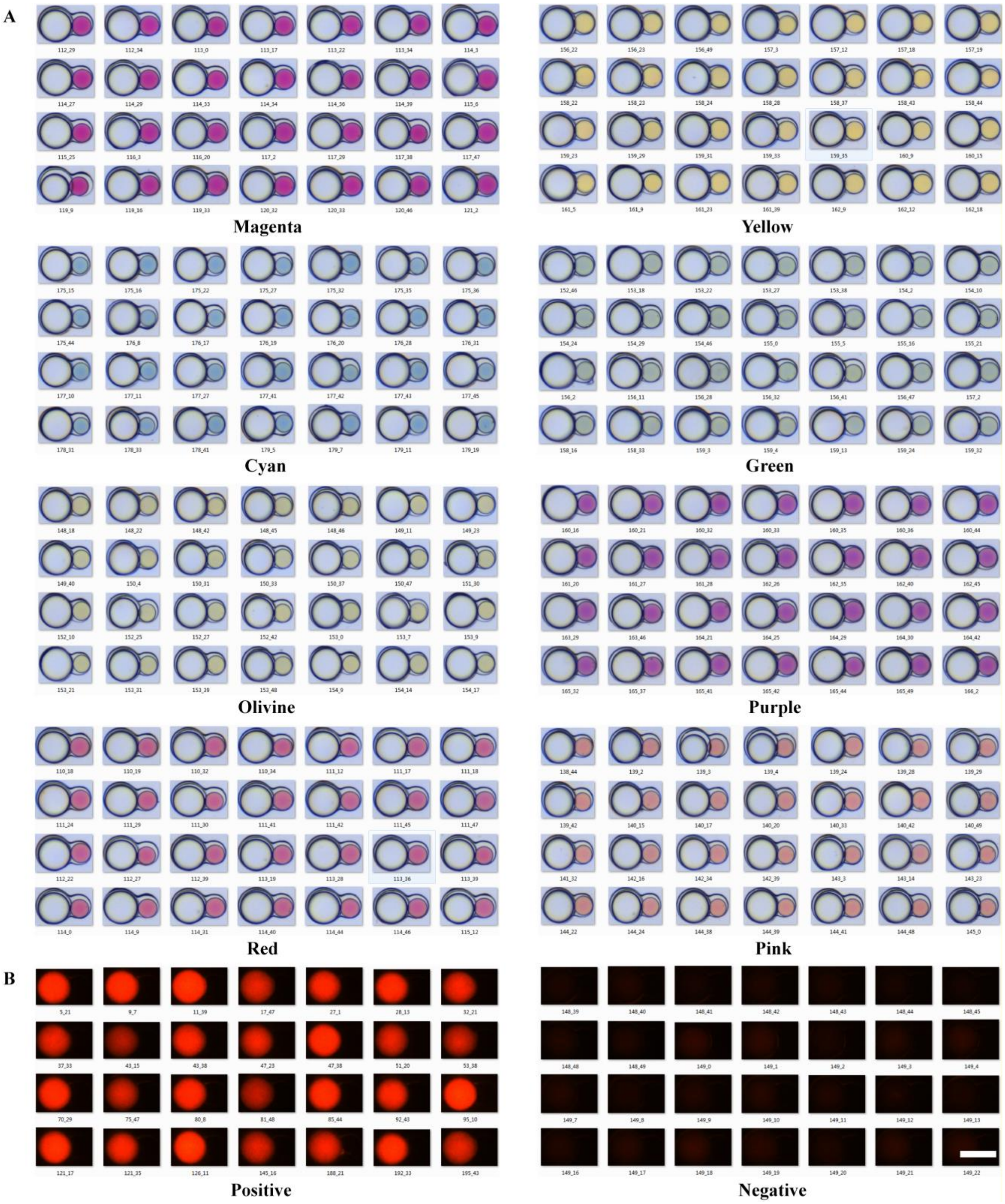

Figure S5. Droplet decoding with the machine learning algorithm. A) After image segmentation, color-encoded primer droplets were classified into 8 groups according to the RGB values. Each microwell containing paired droplets was endowed a specific location information (M_{row}_N_{colume}), and the number of primer droplets in each color was recorded; B) After image segmentation, positive and negative droplets were classified into 2 groups according to the brightness values. Each microwell containing merged droplets was endowed a specific location information (M_{row}_N_{colume}), and the number of positive droplets corresponding to each color code was recorded. (Scale bar=100 μm).

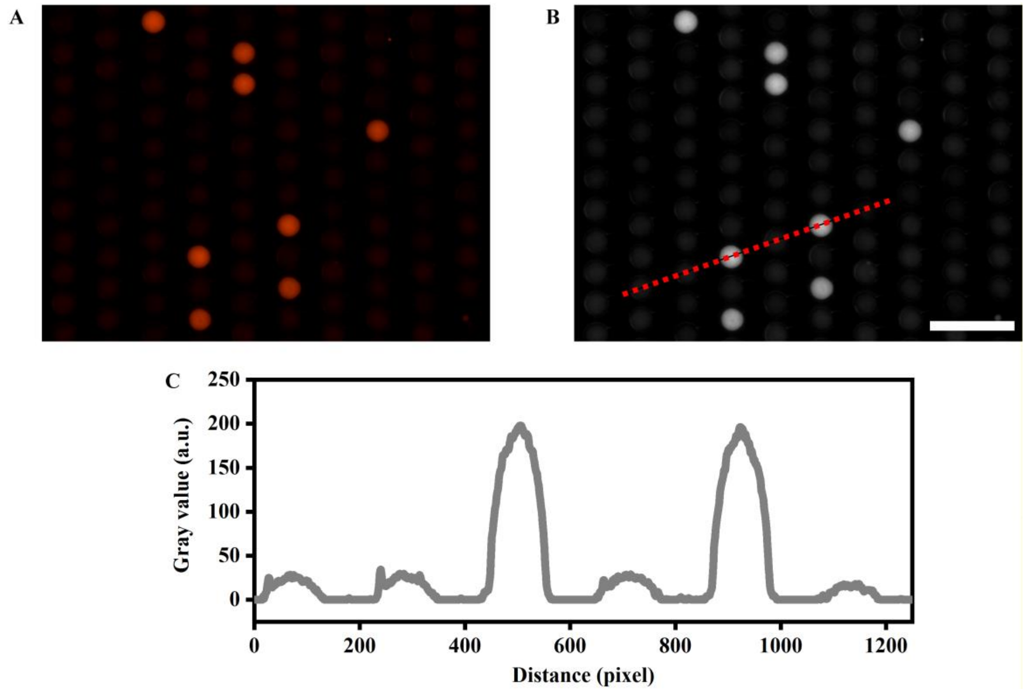

Figure S6. A) Fluorescence microphotograph after the LAMP reaction; B) Transfer the fluorescence microphotograph from RGB into HSB space; C) The linescan presents the fluorescence intensity along the red dashed line: the fluorescence intensity for the microwells containing a DNA template significantly increased, whereas the fluorescence intensity for the microwells without DNA template did not increase. This linescan also verified that there was no cross-contamination between the microwells, since the fluorescence intensity for negative microwells adjacent to the positive ones did not change. (Scale bar=500 μm).

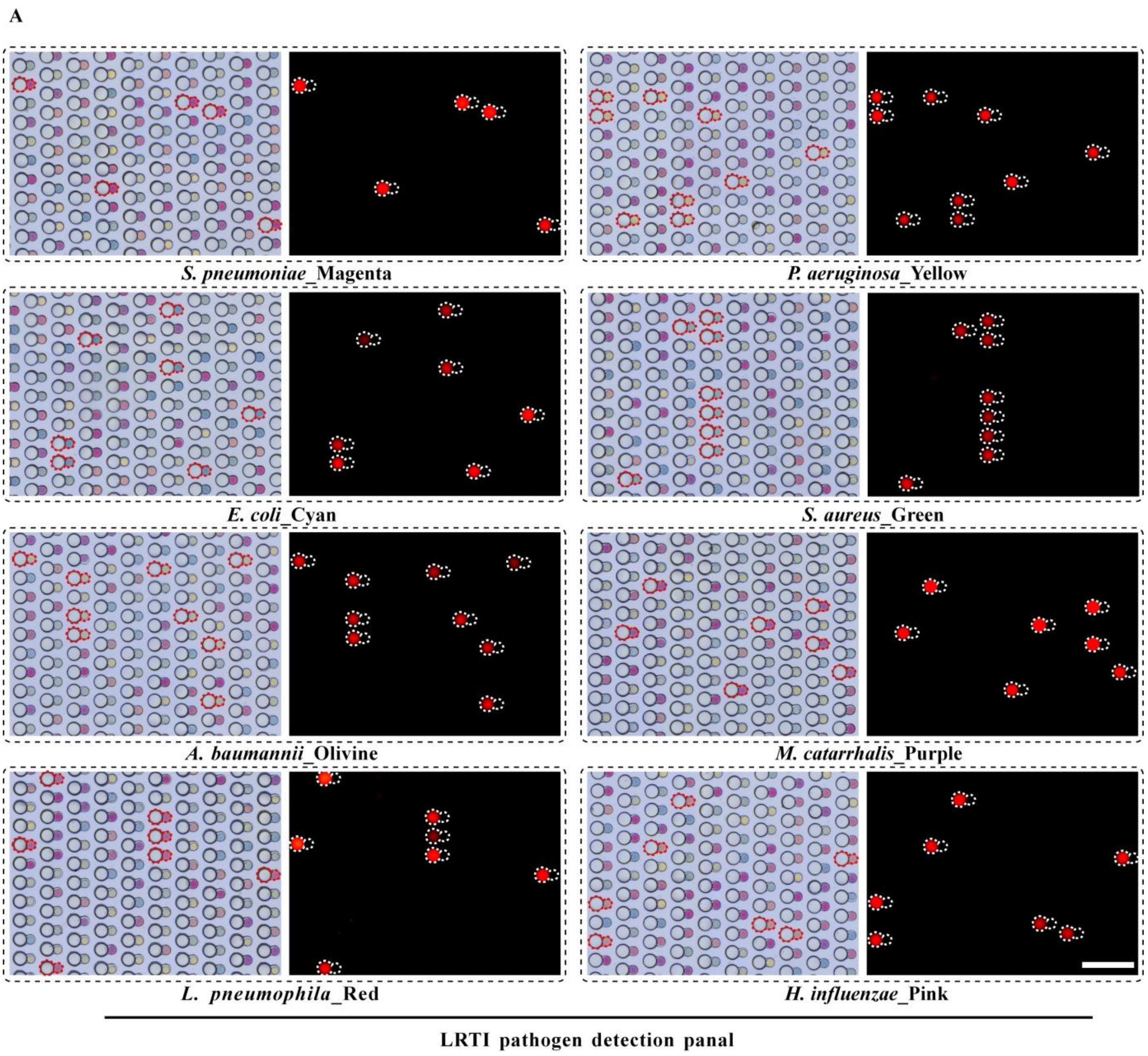
A
S. pneumoniae_Magenta
P. aeruginosa_Yellow
E. coli_Cyan
S. aureus_Green
A. baumannii_Olivine
M. catarrhalis_Purple
L. pneumophila_Red
H. influenzae_Pink
LRTI pathogen detection panal

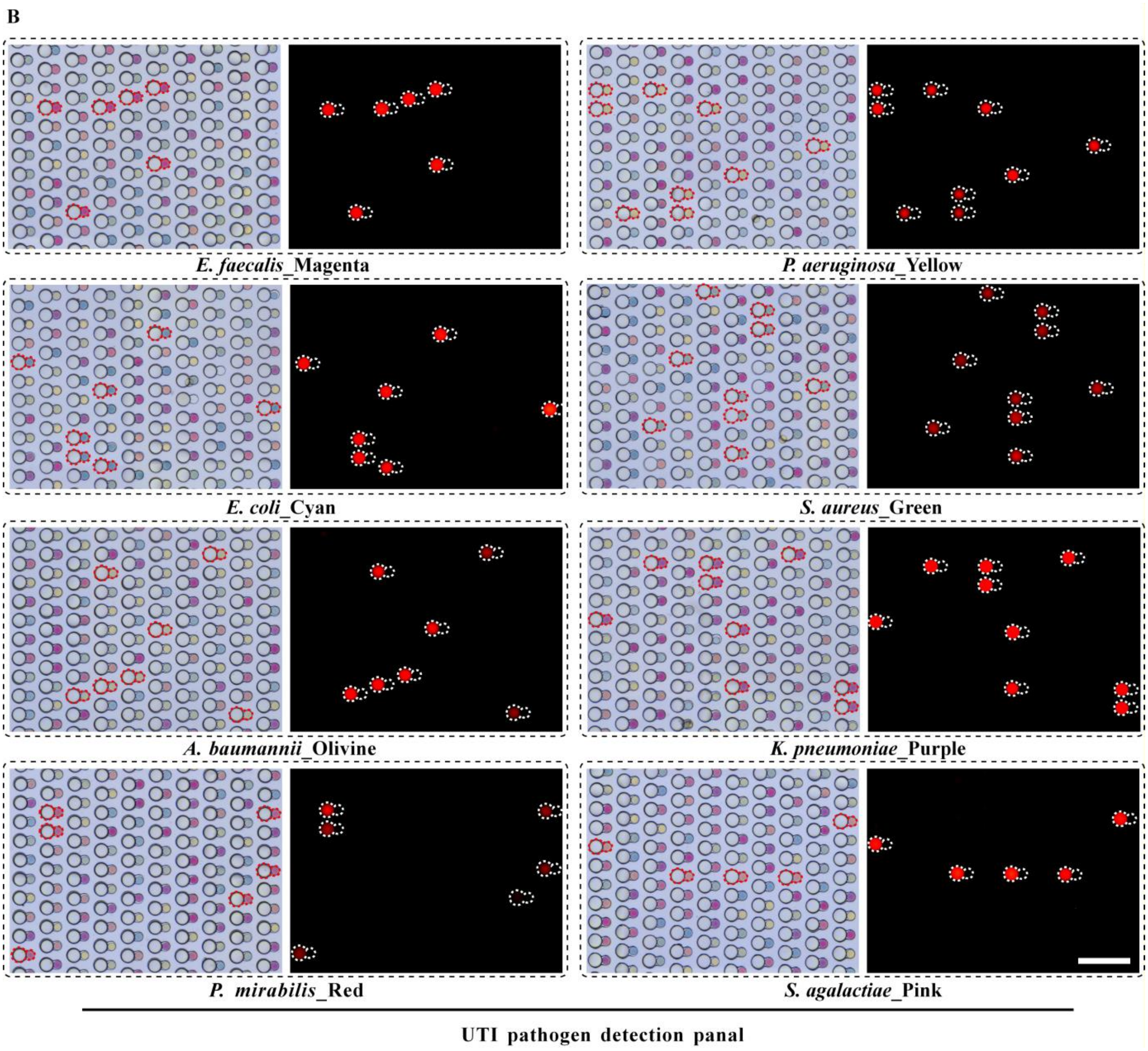

Figure S7. Bright-field and fluorescence microphotographs showing the specificity of the LAMP primers used in the LRTI and UTI detection panels against each input bacterial gDNA. Only the target corresponding merged droplets (dotted calabash-shaped frame) showed increased fluorescence intensity. (Scale bar=500 μm).

Table S1. Color codes and corresponding primers.

Code number	PR (%)	OG (%)	BTB (%)	Color code	Corresponding primer (LRTI detection panel)	Corresponding primer (UTI detection panel)
1	100[a)]	0	0	Magenta	*S. pneumoniae*	*E. faecalis*
2	0	100	0	Yellow	*P. aeruginosa*	*P. aeruginosa*
3	0	0	100	Cyan	*E. coli*	*E. coli*
4	0	40	60	Green	*S. aureus*	*S. aureus*
5	0	70	30	Olivine	*A. baumannii*	*A. baumannii*
6	60	0	40	Purple	*M. catarrhalis*	*K. pneumoniae*
7	50	50	0	Red	*L. pneumophila*	*P. mirabilis*
8	25	75	0	Pink	*H. influenzae*	*S. agalactiae*

[a)]Each column gives the volume fraction (%) of the three indicator dyes.

Table S2. The primer sequences used in the LRTI and UTI detection panels.

Target	Primer sequence (5’-3’)	Target gene
S. pneumoniae	F3: CTG GAG GAA GCA CAC AGA<br>B3: GTC TGG TTT GAG GTA GTA CC<br>FIP: CAC CTT CTT CGT TGA AAT AGT ACC ACT GGT TCG ACA ACT CAG G<br>BIP: GAC AGG CTG GGT CAA GTA CAA TGG ATA AAG GCA TTT GAT ACC<br>LF: AGC GAT TTT CTT CCA GCC<br>LB: CTT AGA CGC TAA AGA AGG CG	*lytA* gene
P. aeruginosa	F3: CTG GCT GCT GTT CTG G<br>B3: CGC TCG TTA GCC TCG T<br>FIP: CTG CGT CTT CGG TAG CGG GGT TGC AGC AGC CAC T<br>BIP: TCA GGC TCG CGC TGA CGA AGT CTG CTG AGC TTT CTG AG<br>LF: TCT TTG GCT TCG AGC AGA CT<br>LB: GCC TAT CGC AAG GCT GAC GAA	*oprI* gene
E. coli	F3: GCC ATC TCC TGA TGA CGC<br>B3: ATT TAC CGC AGC CAG ACG<br>FIP: CAT TTT GCA GCT GTA CGC TCG CAG CCC ATC ATG AAT GTT GCT<br>BIP: CTG GGG CGA GGT CGT GGT ATT CCG ACA AAC ACC ACG AAT T<br>LF: CTT TGT AAC AAC CTG TCA TCG ACA<br>LB: ATC AAT CTC GAT ATC CAT GAA GGT G	*malB* gene
S. aureus	F3: TCG CTT GCT ATG ATT GTG G<br>B3: ACA TAC GCC AAT GTT CTA CC<br>FIP: GTA CAG TTT CAT GAT TCG TCC CGC CAT CAT TAT TGT AGG TGT<br>BIP: TGT TCA AAG AGT TGT GGA TGG TGT ACA GGC GTA TTC GGT T<br>LF: TTG AAA GGA CCC GTA TGA TTC A<br>LB: GAT ACG CCA GAA ACG GTG A	*nuc* gene
A. baumannii	F3: TGT GCC AAT TAA CTT CTT AGC<br>B3: CTT GTA ATA TCG TTA TAG GCG TT<br>FIP: AGT GAA TTC GGT TAT CGT AAG CTC GAA	*adeS* gene

	GCA GCA AAA AAA ATT AGT CAC<br>BIP: AAT GAT ATG GCT CAA AAG CTA GAG GCT TAA CTC ATG TGC GAT GG<br>LF: TAG CAG AGA GGT CGC C<br>LB: CCG TTA AAA ATG CGC AGG	
M. catarrhalis	F3: GAA AGC ACG GCT ACA GAT<br>B3: ACC GAA ACG ACT TTG ACC<br>FIP: CAT GCC ACG AAT GCT GAT AAA TTG AGT GGT TTG TTA CAA GAT GAA CC<br>BIP: GCC ATT GAC CTA AAA ATT GAC AAC GGG GTC TAG CAT AAA GCG A<br>LB: CAC CGC CAA AAC CGA TGG	*copB* gene
L. pneumophila	F3: GTC TTG CGA TTC GAA GCT<br>B3: AAA AAC CAA ATC ACC CAC TT<br>FIP: CCA AGG TTT GCC ATT GAC CAT ATG CAA ATT GCC CCT GAG<br>BIP: GAG AGC GTA TTC CCC TGG ATA TTC ACC AGT AAT CGG CG<br>LF: CGT CCT GTT TCA CTG AAG CC<br>LB: GGC GTG GTT ATT TCA GGT CAG A	*cadA* gene
H. influenzae	F3: TAG AAG GTA ACA CTG ATG AAC G<br>B3: TAC GCT AAC ACT GCA CGA<br>FIP: ACA CCT TTA CCA GCT AAA TAA CCT TTG GTA CAC CAG AAT ACA ACA TC<br>BIP: AGG CAC AGT ATC TTA CGG TGA ATA TGC AGC TTC ATC ATG ACC<br>LF: TGC ACG ACG TTG GCC TAA	*ompP6* gene
E. faecalis	F3: GCC GGA AAT CGA TGA AGA<br>B3: TCC AGC AAC GTT GAT TGT<br>FIP: CAC TTT TTG TTG TTG GTT TTC GCT TTA TTA TCT GCT TGG GGT GC<br>BIP: ATC TGC AGA CAA AGT AGT AAT TGC TCC AAG CTT TTA AGC GTG TC<br>LF: AAA TGC TGC GCC AGC TCG<br>LB: TCC AAT GTG GAA CTT AAA CGT ACC	*azoA* gene[5]
K. pneumoniae	F3: GGA TAT CTG ACC AGT CGG<br>B3: GGG TTT TGC GTA ATG ATC TG	*rcsA* gene[6]

	Fip: CGA CGT ACA GTG TTT CTG CAG TTT TAA AAA ACA GGA AAT CGT TGA GG<br>BIP: CGG CGG TGG TGT TTC TGA ATT TTG CGA ATA ATG CCA TTA CTT TC<br>LB: GAA GAC TGT TTC GTG CAT GAT GA	
P. Mirabilis[a)]	F3: AAA AAA CGC GG**W** TCT GCA<br>B3: AAG ACA GAT AGA GCC AAC G<br>FIP: CTG TCG AGC TAT GGG TAT TAA TCA CTT TTA TTG CGT AAT TGG TTA AAA **R**TC<br>BIP: GTT AGT TGC GCT ATC TTG TGC TTC TTT TGA ACG TGA TAC ATC GGT AGA<br>LF: CCG CCA TAG TAC GTA CTC GCC A	*walR* gene[7]
S. agalactiae	F3: AGA AGC CTT AAC AGA TGT GA<br>B3: CAG GAT AAG TTA AAA CCT TTT GTT C<br>FIP: CAG CTT AGT TAT CCC AAA TCC CAT AGA AGC AAT CAC TTT TTC AAC TCA<br>BIP: ATT CGC ATT TTA GAT CCA TTT GCT TGC CTT TAC ATC GTT AAC TTG AG<br>LF: TGC TTG ACT AAC CTT ATT TGC<br>LB: CAG TTG ATT CAA TTA AAG	*cfb* gene[8]

[a)]Bases in bold letters, W = A or T, R = A or G.

Table S3. Assay time of each step in the mddLAMP assay for detecting the infection-causing pathogens in clinical samples.

Nucleic acid extraction and preparation of LAMP reagents	Template droplet generation and trapping	Primer droplet injection and trapping	Bright-field imaging of paired droplets	Droplet merging	LAMP reaction	Fluorescence imaging of droplets	Image analysis with the machine learning algorithm	Total assay time
~50 min	~10 min	~10 min	~5 min	~1 min	~60 min	~5 min	~2 min	~143 min (<2.5 h)

Table S4. Determination of the limit of blank (LOB) and limit of detection (LOD) based on NTC analysis.

	False positive events											
	Spn	*Pae*	*Eco*	*Sau*	*Aba*	*Mca*	*Sma*	*Hin*	*Efa*	*Kpn*	*Pmi*	*Sag*
NTC-1	0	0	1	0	0	4	0	0	0	0	0	1
NTC-2	0	0	1	0	0	3	0	0	0	0	1	0
NTC-3	0	0	1	0	0	6	0	0	0	0	0	1
NTC-4	0	0	3	0	0	3	0	0	0	0	0	0
NTC-5	0	0	1	0	0	1	0	0	0	0	0	1
NTC-6	0	1	0	1	0	2	0	0	0	0	1	0
NTC-7	0	1	0	0	0	4	0	0	0	0	0	2
NTC-8	0	0	0	0	0	3	0	0	0	0	0	0
NTC-9	0	0	1	0	0	2	0	0	0	0	1	1
NTC-10	0	0	0	0	0	2	0	0	0	0	0	0
False positive ratio (λ_{FP})[a)]	0	0.2	0.8	0.1	0	3	0	0	0	0	0.3	0.6
LOB[b)]	0	2	3	2	0	7	0	0	0	0	2	3
LOD[b)]	3	6	8	5	3	12	3	3	3	3	6	7

[a)] λ_{FP} is the mean number of false-positive events obtained in all NTC experiment; [b)] Using λ_{FP}, we calculated the LOB and LOD for the mddLAMP assays with the statistical analysis method in reference 52.

Table S5. Quantitative detection of pathogens in BALF and urine samples with quantitative bacterial culture and the mddLAMP.

Sample type	Sample ID	Positive/ Negative	Pathogens	Quantitative bacterial culture	mddLAMP
BALF	2530117427	Positive	P. aeruginosa	20,000 CFU·mL^{-1}	2.24×10^{5} cps·mL^{-1}
	3231092881	Negative	ND[a)]	NA[b)]	NA
	3030549630	Positive	*P. aeruginosa*	20,000 CFU·mL^{-1}	6.31×10^{4} cps·mL^{-1}
	3331086645	Negative	ND	NA	NA
	3730694717	Positive	*P. aeruginosa*	>100,000 CFU·mL^{-1}	2.12×10^{5} cps·mL^{-1}
	2531190155	Positive	*S. pneumoniae*	10,000 CFU·mL^{-1}	1.61×10^{4} cps·mL^{-1}
	3230284456	Positive	*M. catarrhalis*	30,000 CFU·mL^{-1}	4.54×10^{4} cps·mL^{-1}
	2330701084	Negative	ND	NA	NA
	2131157301	Positive	*H. influenzae*	30,000 CFU·mL^{-1}	3.25×10^{4} cps·mL^{-1}
	2430062490	Positive	*S. pneumoniae*	20,000 CFU·mL^{-1}	3.86×10^{4} cps·mL^{-1}
	3130694702	Negative	ND	NA	NA
	2531032493	Negative	ND	NA	NA
	2737015416	Positive	*P. aeruginosa*<br>*A. baumannii*	20,000 CFU·mL^{-1}<br>20,000 CFU·mL^{-1}	3.24×10^{4} cps·mL^{-1}<br>2.33×10^{4} cps·mL^{-1}
	4436574559	Positive	*P. aeruginosa*	50,000 CFU·mL^{-1}	7.57×10^{5} cps·mL^{-1}
	4230994584	Negative	ND	NA	NA
	2536806200	Positive	*P. aeruginosa*	>100,000 CFU·mL^{-1}	1.80×10^{5} cps·mL^{-1}
	2931172825	Negative	ND	NA	NA
Urine	57996	Positive	*P. aeruginosa*	>100,000 CFU·mL^{-1}	$>1.55\times10^{7}$ cps·mL^{-1}[c)]
	57393	Negative	ND	NA	NA
	57260	Positive	*E. faecalis*	>100,000 CFU·mL^{-1}	$>1.55\times10^{7}$ cps·mL^{-1}
	57386	Negative	ND	NA	NA
	57388	Positive	*E. coli*	>100,000 CFU·mL^{-1}	$>1.55\times10^{7}$ cps·mL^{-1}
	58251	Positive	*P. aeruginosa*	>100,000 CFU·mL^{-1}	$>1.55\times10^{7}$ cps·mL^{-1}
	57387	Negative	ND	NA	NA
	57394	Negative	ND	NA	NA
	57556	Positive	*E. faecalis*	>100,000 CFU·mL^{-1}	7.85×10^{5} cps·mL^{-1}
	58406	Negative	ND	NA	NA
	57566	Positive	*P. mirabilis*	>100,000 CFU·mL^{-1}	1.54×10^{6} cps·mL^{-1}
	57280	Positive	*E. coli*	>100,000 CFU·mL^{-1}	$>1.55\times10^{7}$ cps·mL^{-1}
	58254	Positive	*K. pneumoniae*	>100,000 CFU·mL^{-1}	$>1.55\times10^{7}$ cps·mL^{-1}
	57997	Positive	*E. coli*	>100,000 CFU·mL^{-1}	$>1.55\times10^{7}$ cps·mL^{-1}
	58407	Negative	ND	NA	NA

	58006	Positive	*E. faecalis*<br>*P. aeruginosa*	>80,000 CFU·mL^{-1}<br>>80,000 CFU·mL^{-1}	2.43×10^{6} cps·mL^{-1}<br>>1.55×10^{7} cps·mL^{-1}
	58408	Negative	ND	NA	NA
	58237	Positive	*K. pneumoniae*	>100,000 CFU·mL^{-1}	>1.55×10^{7} cps·mL^{-1}

[a)]ND, Not detected; [b)]NA, Not applicable; [c)]The theoretical upper detection limit when the target concentration generates only one negative droplet (1 out of 1,250).

Table S6. Comparison of current DNAA multiplexing strategies.

Multiplexing strategy	Multiple fluorescence channels	Real-time monitoring of reaction signals	Co-existence of multiple primer sets/probes	Extensive optimization	Multiplexing expansibility	On-demand flexibility	Reference
Color-based multiplexing	Yes	No	Yes	No	Low	Low	(10, 11)
Amplitude-based multiplexing	No	No	Yes	Yes	Low	Low	(12-15)
Probe mixing-based multiplexing	Yes	No	Yes	Yes	Low	Low	(16, 17)
Amplification curve-based multiplexing	Yes/No	Yes	Yes	Yes	High	Low	(20, 21)
Melting curve-based multiplexing	Yes/No	Yes	Yes	Yes	High	Low	(22-26)
Integration of amplification and melting curves	Yes/No	Yes	Yes	Yes	High	Low	(27, 28)
Padlock probe-based multiplexing	Yes	No	Yes	Yes	High	Low	(18)
Microfluidic chip-based multiplexing	No	No	No	No	High	Low	(8, 29, 30)
Droplet encoding/pairing enabled multiplexing strategy	No	No	No	No	High	High	This work

Movie S1. Trapping of template droplets.

Movie S2. Trapping of primer droplets.

Movie S3. Merging of paired template droplets and primer droplets.

References

[1] G. Nixon, J. A. Garson, P. Grant, E. Nastouli, C. A. Foy, J. F. Huggett, *Anal. Chem.* **2014**, *86*, 4387.

[2] D. T. Chiu, J. Wang, J. P. Staheli, A. Wu, J. E. Kreutz, Q. Hu, J. Wang, T. Schneider, B. S. Fujimoto, Y. Qin, G. S. Yen, B. Weng, K. Shibley, H. Haynes, R. L. Winer, Q. Feng, *Anal. Chem.* **2021**, *93*, 3266.

[3] B. Sun, F. Shen, S. E. McCalla, J. E. Kreutz, M. A. Karymov, R. F. Ismagilov, *Anal. Chem.* **2013**, *85*, 1540.

[4] F. Schuler, C. Siber, S. Hin, S. Wadle, N. Paust, R. Zengerle, F. Von Stetten, *Anal. Methods* **2016**, *8*, 2750.

[5] H. Kato, A. Yoshida, T. Ansai, H. Watari, T. Notomi, T. Takehara, *Oral Microbiol. Immunol.* **2007**, *22*, 131.

[6] D. Dong, W. Liu, H. Li, Y. Wang, X. Li, D. Zou, Z. Yang, S. Huang, D. Zhou, L. Huang, J. Yuan, *Front. Microbiol.* **2015**, *6*, 519.

[7] L. O. Rivoarilala, J. Victor, T. Crucitti, J. M. Collard, *BMC Infect. Dis.* **2021**, *21*, 1037.

[8] K. Kimura, H. Yanagisawa, J. I. Wachino, K. Shibayama, Y. Arakawa, *Jpn. J. Infect. Dis.* **2013**, *66*, 546.
